# Biomechanical analysis of the annular ligament in Monteggia fractures using finite element models

**DOI:** 10.1186/s13018-015-0170-3

**Published:** 2015-03-04

**Authors:** Jiangwei Tan, Mingzhang Mu, Guangjun Liao, Yong Zhao, Jianmin Li

**Affiliations:** Department of Spinal Surgery, Yantai Affiliated Hospital of Binzhou Medical University, Yantai, 264100 China; Department of Orthopaedic Surgery, Yantaishan Hospital, No. 91 Jiefang Road, Yantai, 264001 China; Department of Orthopaedic Surgery, Qilu Hospital, Jinan, 250012 China

**Keywords:** Monteggia fracture, Annular ligament, Finite element, Biomechanics, Three-dimensional model

## Abstract

**Background:**

The pathogenesis of Monteggia injuries remains controversial. The current study biomechanically explored the pathological changes during Monteggia fractures using finite element analysis.

**Methods:**

Two cadaveric forearm specimens underwent computed tomography in both the prone and supine positions. The images were imported to Mimics to construct three-dimensional images. The obtained models of the annular ligaments were assembled onto the bones. Two thin gaps were produced at the proximal third of the ulna to simulate a Monteggia fracture. The models were analyzed mechanically. The initial fracture process was simulated by constraining the distal portions of the radius and ulna and the dorsal fracture sites of the ulna. The mechanical changes of the annular ligament in the two positions were observed and compared.

**Results:**

In the prone position, the maximum *Z*-axial displacement of the annular ligament was close to that along the *Y*-axis, although with a significant difference (*P* < 0.01). In the supine position, the *X*-axial displacement dramatically increased (*P* < 0.01), while it was noticeably decreased along the *Z*-axis (*P* < 0.01).

**Conclusions:**

Biomechanical changes may partially explain the pathological changes in the annular ligament during Monteggia fractures; longitudinal displacement of the radial head causes it to slip out of the annular ligament while the ligament remains intact.

## Background

The Monteggia fracture was originally described as a fracture of the proximal third of the ulna with an anterior dislocation of the radial head. However, due to the common characteristics of several types of upper extremity injuries, the definition of the Monteggia fracture has now been expanded to include all ulna fractures accompanied by radial head dislocation regardless of the location of the fracture or the direction of the dislocation. This type of fracture is usually observed in pediatric patients between 7 and 10 years of age. Pediatric surgeons have sought to understand the mechanism of this fracture because a missed diagnosis or chronic dislocation of the radial head poses treatment challenges.

The pathogenesis of the Monteggia injury remains controversial. Evens described it as a fracture of the ulna with continued pronation that causes leveling out of the radial head [1]. He produced the Monteggia injury in postmortem specimens by applying an excessive pronation force while the ulna was fixed in a vice. Tompkins thought that a fall may cause a pull of the biceps brachii in a hyperextended arm, dislocating the radial head while leaving the ulna to bear the body weight alone, which causes the fracture [2].

In our previous clinical study, we reported that the annular ligaments were intact in pediatric patients with type I and type III Monteggia fractures, with transverse ruptures of the joint capsule at the lower margin of the ligament [3]. Most of the annular ligaments were interposed in the radiohumeral joint even though the radiographs showed a reduction of the radial heads. Based on surgical observations, we hypothesized that the radial head was forced out of the annular ligament by traction rather than by a transverse force that would possibly cause a ligament rupture in adults or a radial head fracture in children.

To prove this hypothesis and to explore the mechanical explanation for the pathology, we designed this biomechanical study using finite element analysis (FEA), which is widely accepted as the most practical and reliable method of analyzing mechanical structures in the field of engineering. Recent developments in computer technology, in both hardware and software, have promoted its medical use. Many studies that apply FEA to the analysis of bones, such as the tibia [4], femur [5], and pelvis [6,7], or to joints [8,9] have been released, and stress or strain distribution patterns in various situations have been reported. However, to the best of our knowledge, there have been no studies applying FEA to Monteggia fractures reported in the current literature.

## Materials and methods

### Construction of finite element models

Two forearm specimens were obtained from the cadaver of a patient who died in his 40s. The specimens were put in prone and supine positions for computed tomography (CT; Philips Company, Holland) scanning (voltage, 120 KV; slice thickness, 0.67 mm). The obtained images were stored in the Digital Imaging and Communications in Medicine (DICOM) format, adjusted according to the bone window in the CT workstation, and then transferred to Mimics (Materalise Company, Belgium), where three-dimensional images were constructed, smoothed, and meshed superficially. The current study was approved by the Ethical Committee of Yantaishan Hospital.

**Figure 1:**
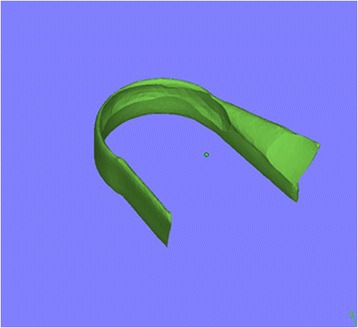
**The constructed annular ligament model after Boolean subtraction.**

**Figure 2:**
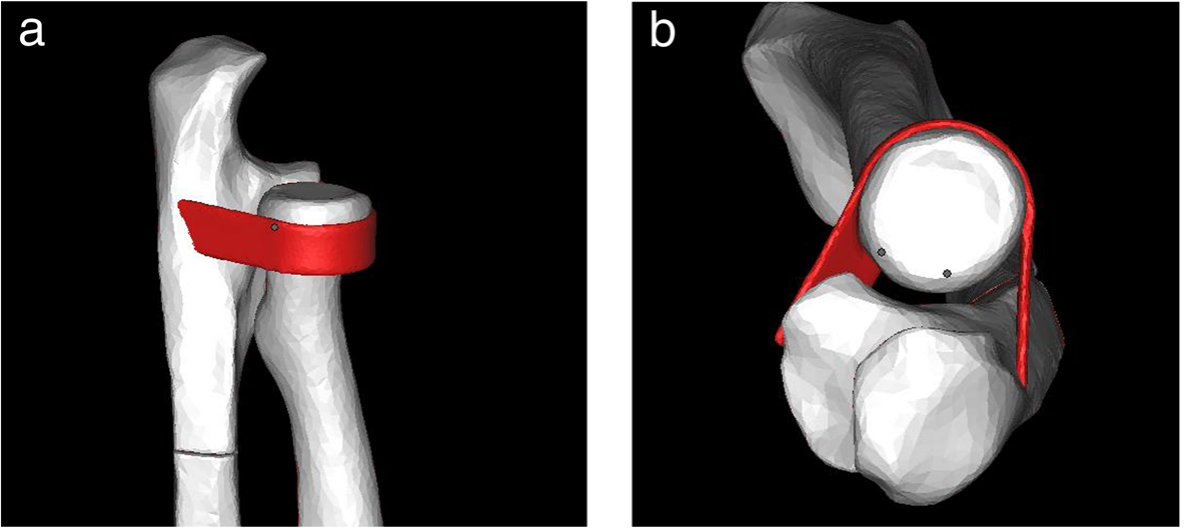
**The combination model of the annular ligament and the bones. (a)** Horizontal view. **(b)** Longitudinal view.

Using the contours of the ulna and radius, a model of the annular ligament was generated. A hollow cylinder and two cuboids with a thickness of 1.5 mm were produced first and then were merged together as a combination. The cylinder contains the radial head and the cuboids touch the sides of the ulna. Boolean subtraction was used to form the ultimate ligament model (Figures 1 and 2). This model was then assembled with the bones to form a bone-ligament combination. A 0.5-mm gap was produced at the proximal third of the ulna to simulate a Monteggia fracture. The combination was re-meshed and optimized, after which it was meshed into tetrahedron units using Patran (NASA, USA). The bones were divided into cortical and cancellous portions according to their density. Mechanical property values were assigned to the bones and the ligaments (cortical bone: Young’s modulus = 10,000 Mpa, Poisson’s ratio = 0.3; cancellous bone: Young’s modulus = 50 Mpa, Poisson’s ratio = 0.26; ligament: Young’s modulus = 50 Mpa, Poisson’s ratio = 0.3) according to the literature [10-12] for a mechanical analysis using Abaqus (ABAQUS, USA).

To accurately capture the three-dimensional (3D) geometry of the bones and the ligament, mesh refinement was performed for the areas with high stress gradients to systematically increase the mesh density until a desired level of accuracy was achieved, whereas preliminary model processing was performed to identify the areas with high discretization errors.

The cortical and cancellous bones were assumed to be linearly isotropic and homogeneous in elements, whereas the ligament was nonlinear because it only undergoes traction forces [10]. The results of FEA were verified using a convergence test to guarantee that the obtained numerical model achieved convergent results and that no further mesh refinement was needed.

Two models were ultimately constructed: the forearm in the prone position and the forearm in the supine position. The element and node data are summarized in Table 1.

**Table 1:** **Elements and nodes in models 1 and 2**

	**Nodes**	**Bone elements**	**Ligament elements**
Prone (model 1)	23,630	97,800	10,788
Supine (model 2)	28,411	116,844	12,878

### Boundary conditions and loading

**Figure 3:**
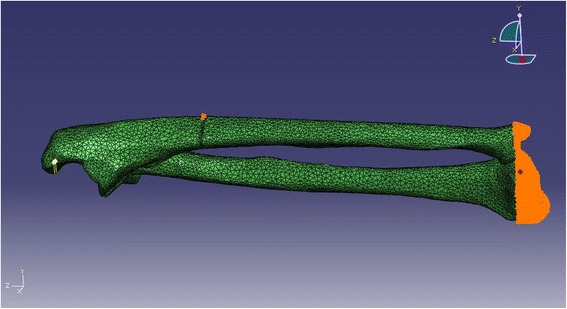
**The fixation points (golden yellow) and loading point (yellow) are shown in different colors.**

The commonly accepted pathogenesis of the Monteggia fracture is that the extended elbow with the olecranon is compressed by the humeral fossa after the distal end of the forearm touches the ground [1,2]. Bearing this in mind, we constrained the distal portions of the radius and ulna and the dorsal fracture sites of the ulna to simulate the momentary status of the ulna immediately after loading. A point load of 100 N was applied to the tip of the olecranon along the *Y*-axis in both models (Figure 3). Because the displacement of the ligament is the focus of this study, we did not have to find the yield point of the ligament. The point load selection was the result of several experiments with our model; with this load, the displacement speed of the bone and the ligament is smooth and proper for animated observations.

### Observation index

The morphological changes in the annular ligament were observed through animations. Thirty consecutive nodes were selected from the anterior portions of both annular ligaments for statistical analysis. The selected nodes were not from the adhesive area of the ligament. The displacement ratios along the *X*-, *Y*-, and *Z*-axes in the two models were demonstrated and analyzed in clinical scenarios.

### Statistical analysis

The displacements were expressed in terms of means and standard deviations. Statistical comparisons were performed using SPSS 11.0 software (USA). Paired *T*-tests were used to compare the displacements of the ligament nodes between axes. An independent sample *T*-test was used to compare displacements between the two models. *P* < 0.01 was regarded as statistically significant.

## Results

**Figure 4:**
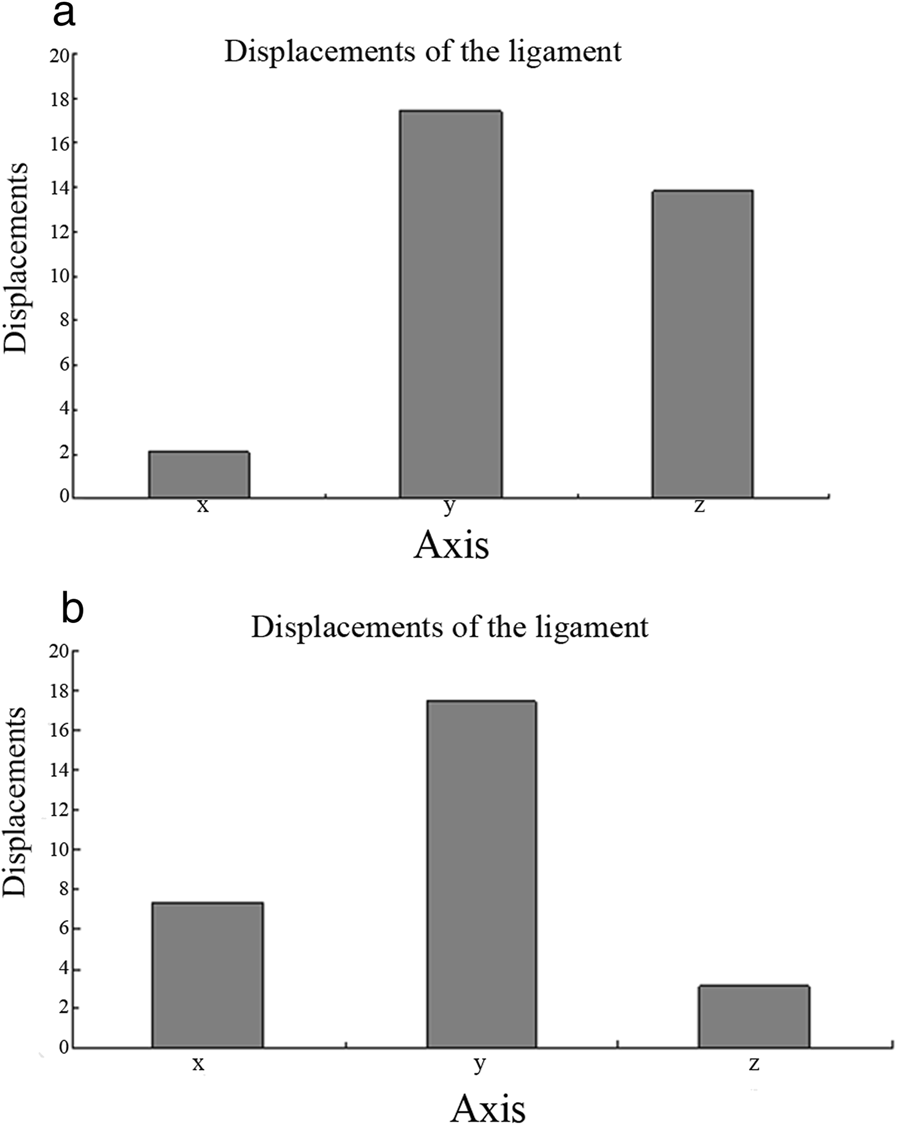
**The displacements of the ligament nodes on three axes (mm). (a)** Model 1, prone. **(b)** Model 2, supine.

The displacement ratios along the three axes are shown in Figure 4a, b. In both models, the displacements along the *X*-, *Y*-, and *Z*-axes were all significantly different (Table 2). In the prone position, the maximum displacement of the annular ligament along the *Z*-axis (longitudinal) was close to that along the *Y*-axis (anteroposterior), although they were significantly different (*P* < 0.01). The displacement of the ligament along the *X*-axis (lateral) was comparatively small (*P* < 0.01). In the supine position, the displacement along the *X*-axis dramatically increased (*P* < 0.01), whereas it was noticeably decreased along the *Z*-axis (*P* < 0.01). The overall displacement of the ligament was primarily along the *Y*- and *Z*-axes. Between the two models, the *X*- and *Z*-axis displacements were significantly different (*P* < 0.01), while the *Y*-axis displacements were not significantly different (*P* > 0.01) (Table 3).

**Table 2:** **Comparisons of the ligament displacements in three planes using paired** ***T*** **-tests**

**Plane**	**Prone (model 1)**				**Supine (model 2)**			
	**Mean (mm)**	**Std. deviation (mm)**	***T*** **-value**	***P*** **value**	**Mean (mm)**	**Std. deviation (mm)**	***T*** **-value**	***P*** **value**
*X*-*Y*	15.320	4.196	0.203	0.000	10.091	2.886	0.195	0.000
*X*-*Z*	11.755	3.743	0.175	0.000	14.326	1.597	0.500	0.000
*Y*-*Z*	3.568	0.620	0.320	0.000	4.235	1.431	0.165	0.000

**Table 3:** **Comparisons of the ligament displacements between model 1 and model 2 using T-tests**

**Axis**	**Mean displacement in model 1 (mm)**	**Mean displacement in model 2 (mm)**	***T*** **-value**	***P*** **value**
*X*	2.084	7.340	−0.254	0.000
*Y*	17.407	17.432	−0.000	0.977
*Z*	13.839	3.107	0.154	0.000

**Figure 5:**
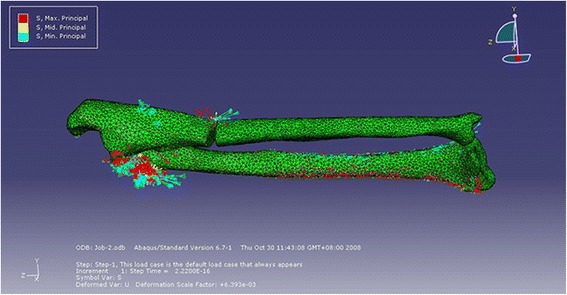
**The stress concentration of the combination after loading.**

**Figure 6:**
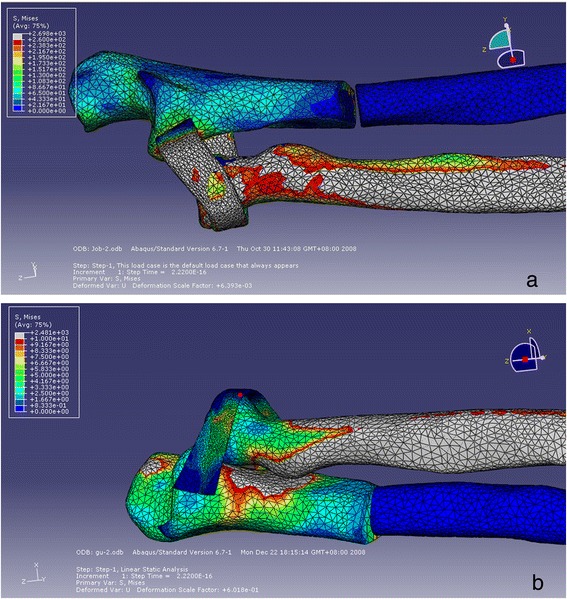
**Quantification of the stress on the bones and the ligament after loading. (a)** Model 1, prone. **(b)** Model 2, supine.

The stress concentration after loading is shown in Figure 5. The picture shows that the stress is concentrated on the ulnar fracture sites and the annular ligament. Although there was no load on the radius, the stress was transmitted to the radius through the annular ligament. The stress distributions and quantification of the two models are shown in Figure 6a, b.

## Discussion

Although it has been frequently discussed in the literature, the mechanism underlying a Monteggia fracture remains controversial. In our previous clinical study, we hypothesized on the basis of surgical observations that the radial head was forced out of the annular ligament by traction rather than by a transverse force [3]. Since the annular ligament pathology findings were first reported, there have been no studies reported in the literature concerning an explanation of these surgical findings. Therefore, we designed this biomechanical research study, which demonstrated the mechanical changes occurring in the annular ligament in a typical Monteggia fracture using FEA to further elucidate the pathogenesis of this fracture. We believe that a sound mechanical explanation for this specific fracture-dislocation may provide a foundation for its treatment in clinical practice.

FEA has two advantages. First, it can avoid the interference caused by individual differences in bone density, and second, it more clearly shows the changes in the annular ligament when compared with entity model analysis. However, a disadvantage of FEA is the difficulty in simulating the material property of an entity accurately. To overcome this shortcoming, we presented our results in terms of ratios.

The most difficult part of FEA model generation lies in the construction of the annular ligament. Because the annular ligament is too delicate to be extracted and because obtaining an intact ligament model from CT scans is impractical, we artificially constructed it according to the contour of the radial head. We then assembled it onto the bones to form a bone-ligament combination model. Because the normal friction property of the annular ligament was changed into a gluey property, we successfully simplified the analytical complexity. Accordingly, the stress on the ligament was indirectly expressed by its displacement.

In this study, we simulated the entire fracture process by constraining the distal radius and ulna in the model. Although muscles definitely play a role during the process of loading, we omitted their effect because this study focused on the mechanical pathological changes in the annular ligament, which were separate from the fracture site. More specifically, we studied what would happen after ulnar yielding but before the yielding of the bone.

The results showed that, in the prone position, the overall displacement of the annular ligament was primarily along the *Y*- and *Z*-axes with the maximum *Z*-axial displacement close to the *Y*-axial displacement even though they were significantly different (*P* < 0.01), whereas the displacement along the *X*-plane was comparatively minor (*P* < 0.01). In the supine position, the displacement on the *X*-plane dramatically increased (*P* < 0.01), whereas displacement on the *Z*-plane noticeably decreased (*P* < 0.01). These findings suggest that the radial head is less likely to dislocate from the lower edge of the ligament while in a supine position than in a prone position. This is consistent with the report by Evens that excessive pronation plays an important role in the production of type I fractures [1]. The pathogenesis of a type III Monteggia fracture is basically the same as that of a type I fracture except for a different vector force [3]. Although this does not prove that pronation of the forearm is the direct cause of this type of injury, we have a reason to speculate that pronation also plays a role in type III fractures because we found very similar annular ligament changes during surgical observations.

In surgery, we found that as the angulation of the ulna fracture increases, the radial head begins to move distally apart from the annular ligament and finally detaches from the ligament; a prone position of the forearm will facilitate the process of this detachment [3]. Based on findings from clinical practice and the FEA results of this study, we conclude that in a Monteggia fracture, the radial head slips out of the annular ligament by a traction force rather than bursting out of the ligament by a transverse force.

This study has some limitations. First, FEA cannot perfectly simulate the real situation that occurs during injury due to the complexity of human anatomic structures and the imperfections of the FEA software. With the development of computer-aided engineering software, this shortcoming of FEA may be overcome. Second, it is difficult to obtain sufficient fresh specimens (without preservative). The properties of the intersection of the annular ligament and capsule could be greatly affected by preservatives. Although the mechanical property values were assigned to the bone and the ligament according to the literature, the final property values could be affected by the thickness of the cortical and cancellous bones in different specimens. Therefore, it would be theoretically better to increase the number of specimens to obtain mean values for the structural indices.

## Conclusions

In this study, we constructed a FEA model of the ulna and radius in combination with the annular ligament. A Monteggia fracture was simulated by constraining both the distal portions of the radius and ulna and the dorsal fracture sites of the ulna. The greater *Z*-axial displacement of the annular ligament in the prone position than in the supine position explained the longitudinal stretch of the radial head out of an intact annular ligament that was observed during surgery. This clear explanation of annular ligament pathology may guide surgeons when they are treating a Monteggia fracture-dislocation.

## Abbreviations

FEA: Finite element analysis
CT: Computed tomography
DICOM: Digital Imaging and Communications in Medicine
3D: Three-dimensional
